# On Exostosis and Some Other Diseases of the Teeth and Their Consequences

**Published:** 1853-04

**Authors:** 


### ARTICLE XV.

*On Exostosis and some other Diseases of the Teeth and their Consequences.*

He commenced by drawing attention to the healthy structures perceived in a section of a tooth when viewed microscopically. He demonstrated by an ingenious set of models, how and why the enamel consisted of hexagonal crystals, and how it was unlikely that any disease should occur after the enamel was once healthily formed, except from mechanical or chemical causes. Caries of a tooth most usually commenced in the enamel, and was a consequence of chemical action produced during fermentation by some portion of food lodging in a groove or indentation in the crown of a tooth; this, and the progress of caries, were shown in a series of preparations. The fact itself had been demonstrated to him by his late friend, Mr. Durance George. Irregularity in the deposit of the enamel was alluded to, such as is frequently seen in the second set of teeth. This might be due to scrofula, fever, or other disease affecting the child at the time of the deposit of new material, or sometimes to the injudicious administration of mercury. In the ivory, or dentine, no exostosis ever occurred: the material consisted of tubes too small in diameter to admit a red globule of blood, the vitality of the dentine being maintained by endosmosis and exosmosis. When, from disease, the tubes were filled with an earthy or chalky material the teeth soon became fractured and destroyed. The cementum, or last structure seen in the section, was similar to bone, with the exception that Haversian canals had very rarely been detected in it. This cementum surrounded the roots of a tooth, and was very frequently the source of much disease, when exostosed, as is often the case. The exostosis was of two kinds—the ivory and the common osseous exostosis. The latter differed in structure from the healthy cementum by being apparently formed round the fang in laminæ, and the lacunæ or corpuscles being regularly deposited in rings around the root, whereas in healthy cementum the lacunæ are scattered irregularly. An interesting case was related of abscess of the antrum, occurring from an ivory exostosis pressing on the membrane of that cavity; the bony floor, if it had ever existed, having been absorbed by pressure. The result was favorable, the tooth having been extracted with much caution by Mr. Fox. The after-treatment was that usually resorted to in similar cases of abscess of the antrum. An interesting specimen of cemental exostosis was shown as having caused an abscess in the jaw of the elephant Chuney, who some years back was shot, under a mistaken notion of the cause of his fury, at Exeter Change. Reference was made to the consequence of teeth—especially the dentes sapientiæ—taking a wrong direction whilst being erupted. Cases were quoted in illustration of this, as well as of other points alluded to in the paper, and a variety of preparations and drawings were exhibited to the society.

